# CASE REPORT Acute Compartment Syndrome of the Forearm Following Blood Gas Analysis Postthrombolysis for Pulmonary Embolism

**Published:** 2013-03-07

**Authors:** Kamal Bisarya, Samuel George, Sam El Sallakh

**Affiliations:** ^a^Department of Orthopaedic Surgery, The Witthington Hospital, London; ^b^Department of Burns and Plastic Surgery, Morriston Hospital, Swansea; ^c^Department of Orthopaedic Surgery, The Whittington Hospital, London, United Kingdom

## Abstract

**Objectives:** Acute compartment syndrome is an important condition with potentially serious consequences if not diagnosed and treated promptly. This report highlights a case of acute compartment syndrome of the forearm after radial artery blood gas analysis in a patient who had been thrombolyzed for a pulmonary embolus. **Methods/Case Report:** We present a case of a 54-year-old lady, admitted and treated for a pulmonary embolism with tenecteplase for thrombolysis. As per routine management, she had taken an arterial blood gas sample, which caused hematoma in the wrist and a few hours later developed pain and a tense right forearm being diagnosed with compartment syndrome. **Results:** She underwent fasciotomies and subsequent split skin grafting. We discuss the different etiologies of compartment syndrome, clinical signs, and available investigations as well as immediate and definitive management options including fasciotomy techniques. We present the latest literature on the subject and extract valuable learning points from this case. **Conclusions:** With the common use of thrombolysis for the management of a myocardial infarction or pulmonary embolus, compartment syndrome is an uncommon but potentially associated problem. Furthermore, with blood gas sampling being part of daily clinical practice and a potential cause of this condition, the compartment syndrome becomes iatrogenic and potentiates serious litigation. As many junior doctors are performing blood gas analysis postthrombolysis, they need to assess patients adequately and realize the risk of possible sequelae such as compartment syndrome in this group and inform patients of such complications.

Acute compartment syndrome is an important condition with potentially serious consequences if not diagnosed and treated promptly. This report highlights a case of acute compartment syndrome of the forearm after radial artery blood gas analysis in a patient who had been thrombolyzed for a pulmonary embolus.

## CASE STUDY

M.S. was a 54-year-old lady who was admitted with acute shortness of breath and pleuritic chest pain. A computed tomography pulmonary angiogram (CTPA) confirmed a pulmonary embolus in a segmental branch of the pulmonary artery, and as the patient was compromised she was thrombolyzed with tenecteplase. Postthrombolysis and while on enoxaparin (Clexane), the patient had a blood gas sample taken from the right radial artery. A few hours later, it was noted that M.S. complained of pain on passive movement of the wrist and fingers, and a painful and tense right forearm with altered sensation and tingling in the radial 3½ right fingertips. On examination, the flexor surface of the forearm was tense and swollen, with inability to actively flex or extend the fingers or the wrist. A suspicion of early compartment syndrome was made, and therefore the evening dose of low-molecular-weight heparin was omitted and an emergency fasciotomy was done. The findings were of hematomas on the distal and proximal aspect of the right forearm, tightness of the deep fascia sheaths along its whole length with “bursting” of the muscle on release, scattered partial necrosis of the flexor groups, and compression of the median nerve in the carpal tunnel. Postoperatively, she made an uneventful recovery and subsequently a split skin graft to the right arm was performed by plastic surgeons.

## DISCUSSION

Compartment syndrome is a problem characterized by raised tissue pressure within a closed osteofascial compartment, which compromises blood flow to muscles and nerves and therefore has the potential to cause irreversible damage to the contents of the compartment. Muscles have functional impairment after 2 to 4 hours of injury, and within 4 to 12 hours muscle infarction occurs.^1^ Prolonged muscle ischemia and necrosis can lead to scarring and Volkmann's contracture. Cellular destruction may also lead to the release of myoglobin into the circulation, and later rhabdomyolysis, renal failure, and even death may result.

There are many etiologies of compartment syndrome including trauma (eg, fractures and burns), external restriction of the compartment (eg, tight casts or dressings), and internal increase in compartment volume (eg, hemorrhage and compartment fluid injection including thrombolytic therapy^2^^,^^3^). Although acute compartment syndrome is a rare and not well-described sequelae of arterial blood gas sampling, the risk is real and actually, the first reported case was in 1978.^4^ There are a number of reasons why arterial blood gas sampling causes compartment syndrome. This can occur in patients with or without a bleeding diathesis. In most cases of compartment syndrome due to arterial blood gas sampling, this is associated with concurrent anticoagulant therapy although there have also been cases in patients with other causes of bleeding diathesis such as chronic renal failure^5^ and after repeated sampling causing false aneurysms.^6^

Symptoms are due to lack of oxygenated blood and waste product removal. The patient often complains of pain out of proportion to the initial injury that is worsened by passive stretch of the fingers. The patient may also describe a tense feeling, and on examination, there may be a firm wooden feeling on deep palpation. Less reliable signs include paraesthesia or numbness—especially over the fingertips (however, 2-point discrimination is a more reliable early test). The presence of a radial pulse does not exclude the possibility of this condition and actually the finding of pulselessness and pallor are both late findings. A motor deficit is an even later sign.^1^

Various investigations can aid diagnosis. Creatine kinase reflects the level of muscle necrosis—serial rising levels indicate progression of the condition and very high levels indicate rhabdomyolysis. If rhabdomyolysis is present then kidney function can be assessed via urea and creatinine levels. Needle instruments are available for direct pressure measurement. There are different opinions on the compartment pressures although many surgeons use 40 mm Hg as an indication for fasciotomy. Others believe it is more accurate to relate compartment pressures to the patient's diastolic pressure as patients with higher blood pressures may have higher compartment pressures without a clinical compartment syndrome and vice versa. Authors such as Whitesides et al^7^ advocate a pressure of 20 mm Hg less than the diastolic blood pressure as an indication, whereas McQueen et al^8^ suggest a differential pressure of less than 30 mm Hg between the diastolic and compartment pressures as an indication.^8^ That being said, however, clinical suspicion is ultimately the best indication.

Management involves general, medical, and surgical measures. General measures include placing the arm at the level of the heart and removing any external restricting device (this decreases compartment pressure greater than just releasing one side of the plaster or bivalving alone). Medical management includes making sure associated compounding factors (eg, concurrent hypertension and anemia) are corrected, and corresponding rhabdomyolysis, hypovolemia (bleeding), and acidosis are treated with fluids. The role of mannitol has been discussed in the literature, and it has shown promising results in an animal model in reducing the risk of reperfusion injury by reducing compartment pressures due to it's osmotic activity.^9^ The data on humans also suggest that hypertonic mannitol may have a protective effect on ischemia-reperfusion injury by reducing edema but its role is still under investigation and more trials are needed before this is put into practice.^10^^,^^11^ However, the treatment of choice for compartment syndrome is early emergency decompression via fasciotomy.

Intraoperatively no tourniquet is used (although it can be used if profuse bleeding is anticipated^12^). Within the forearm, elevated pressures more commonly occur in the volar compartment than in the volar and dorsal together, and rarely in the dorsal alone. Fasciotomies can be performed via a volar or dorsal approach or both, but as the 3 compartments of the forearm (superficial and deep volar, dorsal, and the compartment containing the mobile wad of Henry) are interconnected, fasciotomies of all 3 compartments are often unnecessary.^1^ There are 3 main types of operative technique in the forearm—a volar Henry approach, a volar ulnar approach, and a dorsal approach. In this case, a volar Henry approach was used. The incision is left open with a sterile moist dressing placed on top. The arm is then elevated for 24 to 48 hours postsurgery. After 5 days, delayed primary closure of the skin is performed, but if this is not possible, then a split thickness skin graft is applied—like in our case. If heparin needs to be continued while the incision is left open, then a homograft (allograft/cadaveric skin) could be applied to the fasciotomy wound site to control ongoing blood loss. The homograft can be removed when primary closure of the fasciotomy site is needed.^12^ If diagnosed late, fasciotomy is of no benefit (and is probably contraindicated after day 3 or 4) as this increases the risk of infection of the necrotic muscle.^1^

## LEARNING POINTS

With the common use of thrombolysis for the management of a myocardial infarction or pulmonary embolus, compartment syndrome is an uncommon but potentially associated problem. Furthermore, with blood gas sampling being part of daily clinical practice and a potential cause of this condition, the compartment syndrome becomes iatrogenic and potentiates serious litigation, with recent settlements amounting to nearly half a million US dollars.^13^^,^^14^ As many junior doctors are performing blood gas analysis postthrombolysis, they need to asses patients for being at high risk and realize the risk of possible sequelae^12^ like compartment syndrome in this group and may need to inform patients of such complications.^15^

## Figures and Tables

**Figure 1:**
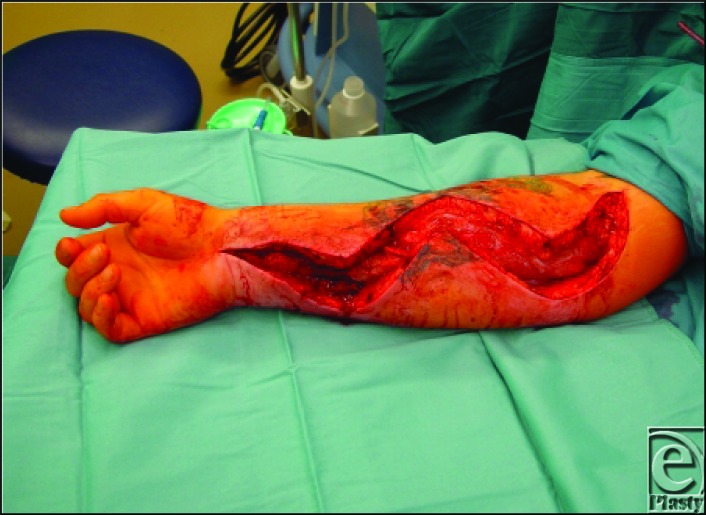
Forearm post compartment decompression.^16^
